# Supplementary material for: Exploring the relationships between pre-pregnancy BMI, gestational weight gain, and nutritional intake: a real-world investigation in Shandong, China
Source: PeerJ. 2024 Mar 22;12:e17099. doi: 10.7717/peerj.17099 (PMC10962341; doi:10.7717/peerj.17099)
Supplement: Supplemental Information 2 [file peerj-12-17099-s002.docx]

**Table S1** Classification of food items in CHDI-P

| Food Categories | Definition and representative foods |
| --- | --- |
| Whole grains | Grains that have not been finely processed or which have been milled, crushed, or laminated but still retained the whole husk, aleurone layer, endosperm, embryo and natural nutrients of the whole grains. Dietary fibre content/carbohydrate content ≥ 0.1, such as roughage rice, oats, corn, millet, buckwheat, etc. |
| Mixed beans | Mixed beans have higher carbohydrate content than soybeans, with about 50~60% starch, such as red beans, kidney beans, mung beans, peas, chickpeas, broad beans, etc. |
| Tubers | Potatoes, sweet potatoes, taro, yams and cassava, etc. |
| Refined grains | Wheat products or flours removed bran and germ with low fibre content. Dietary fibre content/carbohydrate content < 0.1, such as refined rice, refined flour, etc. Products made from refined flour such as breads, noodles, cookies and baked goods/desserts were also included. |
| Dark-colored vegetables | Dark-colored vegetables are dark green, red, orange and fuchsia vegetables rich in β-carotene. Dark green vegetables such as spinach, leeks and broccoli, etc. Orange and red vegetables such as carrots, tomatoes and pumpkins, etc. Fuchsia vegetables such as purple cabbage. |
| Fruits | Fresh and whole fruits such as citrus, pome fruits, berry, melons, drupe fruits, etc. |
| Red meats | Fresh, unprocessed meat, including the muscle and offal of pigs, cattle, sheep, etc. |
| Processed meats | Meat products processed with raw livestock and poultry, including pickled products, sauce braised products, barbecue products, sausage, ham and canned meat, etc. |
| Fish and seafood | Freshwater fish, marine fish, shellfish, mollusks, etc. |
| Eggs | Eggs and their products |
| Dairy | Milk, Yogurt, Formula milk powder |
| Soybeans | Soybeans include green beans, black beans and soybeans. Soybeans products are divided into non-fermented and fermented soybean products. Non-fermented products include soybean milk, tofu, dried tofu, etc. Fermented products include fermented bean curd, fermented black beans, etc. |
| Nuts | Walnut, peanut, melon-seeds, almond, etc. |
| Fried foods | Cooked foods that are fried at high temperatures, such as deep-fried dough sticks, fried bread, etc. |
| Sugary beverages | Beverages containing more than 5% sugar, such as cola, juice with added sugar, etc. |
| Alcohol | Drinks such as beer, wine, etc. that can make people drunk. |
| Iodized salt | Iodized salt is sodium chloride containing potassium iodate. |
| Cooking oil | Vegetable oil such as canola oil, soybean oil, etc. Animal oil such as lard, beef tallow, etc. |
| Folic acid supplements | Folic acid, 5-methylmethohydrofolate (5MTHF), or leucovorin in tablet, capsule, dispersible tablet, or liquid form. |

 Food categories comes from the Chinese dietary guidelines for Pregnant Women and the Chinese balanced dietary pagoda for pregnant women.

 The definition of food categories comes from the Chinese dietary guidelines for Pregnant Women and the China food composition tables (6th edition).

Abbreviations: CHDI-P, Chinese Healthy Diet Index for Pregnancy.
