# Supplementary material for: Exploring the relationships between pre-pregnancy BMI, gestational weight gain, and nutritional intake: a real-world investigation in Shandong, China
Source: PeerJ. 2024 Mar 22;12:e17099. doi: 10.7717/peerj.17099 (PMC10962341; doi:10.7717/peerj.17099)
Supplement: Supplemental Information 3 [file peerj-12-17099-s003.docx]

**Table S2** Chinese Healthy Diet Index for Pregnancy (CHDI-P) components and standard for scoring

| **Components** | | **Score** | | | | | |
| --- | --- | --- | --- | --- | --- | --- | --- |
| **Diversity** | | | | | | | |
| Category of food | | Recommended number of species/d | | | Range of score | | |
| Grains, Tubers and Mixed beans | | 3 | | | 0-3 | | |
| Vegetables and Fruits | | 4 | | | 0-4 | | |
| Meat, Poultry, Fish and Eggs | | 3 | | | 0-3 | | |
| Dairy, Soybeans and Nuts | | 2 | | | 0-2 | | |
| Total | | 12 | | | 0-12 | | |
| **Adequacy** | | | | | | | |
|  | | Unit | Base Score (BS) | | | Weighting | Range of score |
|  |  |  | 0 | 1.5 | 3 |  |  |
| Whole grains/Mixed beans | | g/d | 0 | (0,50) | ≥50 | 2BS | 0,3,6 |
| Tubers | |  | 0 | (0,50) | ≥50 | 1/3BS | 0,0.5,1 |
| Poultry | |  | 0 | (0,40) | ≥40 | BS | 0,1.5,3 |
| Fish and seafood | |  | 0 | (0,40) | ≥40 | BS | 0,1.5,3 |
| Eggs | |  | 0 | (0,50) | ≥50 | BS | 0,1.5,3 |
| Dairy | |  | 0 | (0,300) | ≥300 | BS | 0,1.5,3 |
| Soybeans | |  | 0 | (0,15) | ≥15 | BS | 0,1.5,3 |
| Animal liver | |  | 0/>50 | (0,25) | [25,50] | BS | 0,1.5,3 |
| Animal blood | |  | 0/>50 | (0,25) | [25,50] | BS | 0,1.5,3 |
| Red meats | |  | 0/>100 | (0,50) | [50,100] | BS | 0,1.5,3 |
| Dark-colored vegetables | |  | 0 | (0,200) | ≥200 | 2BS | 0,3,6 |
| Fruits | |  | 0/>350 | (0,200) | [200,350] | BS | 0,1.5,3 |
| Marine algae | |  | 0 | (0,20) | ≥20 | BS | 0,1.5,3 |
| Iodized salt | |  | 0/>15 | (6,15] | (0,6] | BS | 0,1.5,3 |
| Nuts | |  | 0 | (0,10) | ≥10 | BS | 0,1.5,3 |
| Folic acid supplements | | μg/d | 0 | (0,400) | ≥400 | 2BS | 0,3,6 |
| **Limitation** | | | | | | | |
|  | Unit | Base Score (BS) | | | | Weighting | Range of score |
|  |  | 0 | | 2.75 | 5.5 |  |  |
| Fried foods | g/d | ≥200 | | (0,200) | 0 | BS | 0,2.75,5.5 |
| Sugary beverages |  | ≥400 | | (0,400) | 0 | BS | 0,2.75,5.5 |
| Processed meats |  | ≥50 | | (0,50) | 0 | BS | 0,2.75,5.5 |
| Alcohol |  | >0 | | _ | 0 | BS | 0,5.5 |
| Refined grains |  | <170/>450 | | _ | [170,450] | BS | 0,5.5 |
| Cooking oil |  | 0/>50 | | (25,50] | (0,25] | BS | 0,2.75,5.5 |
