# Supplementary material for: Exploring the relationships between pre-pregnancy BMI, gestational weight gain, and nutritional intake: a real-world investigation in Shandong, China
Source: PeerJ. 2024 Mar 22;12:e17099. doi: 10.7717/peerj.17099 (PMC10962341; doi:10.7717/peerj.17099)
Supplement: Supplemental Information 4 [file peerj-12-17099-s004.pdf]

STROBE Statement—checklist of items that should be included in reports of observational studies

	Item No.	Recommendation	Page No.	Relevant text from manuscript
**Title and abstract**	1	(*a*) Indicate the study's design with a commonly used term in the title or the abstract	1	Exploring the Relationships Between Pre-Pregnancy BMI, Gestational Weight Gain, and Nutritional Intake: A Real-World Investigation in Shandong, China
		(*b*) Provide in the abstract an informative and balanced summary of what was done and what was found	1	This study investigated the associations between gestational weight gain (GWG), pre-pregnancy body mass index (BMI), and prenatal diet quality in pregnant women from Shandong, China……This indicates that greater GWGs do not necessarily align with principles of adequate nutrition.
**Introduction**				
Background/rationale	2	Explain the scientific background and rationale for the investigation being reported	2	The profound association between maternal and newborn health has been acknowledged for an extended period, and endeavors to enhance health outcomes persist……Prior research has examined the prenatal dietary quality of expectant mothers. In the United States, the Healthy Eating

				Index-2010 score for these women stands at a mere 50.7 out of 100
Objectives	3	State specific objectives, including any prespecified hypotheses	2-3	This exploration sought to identify a subgroup particularly susceptible to compromised dietary quality. We posited that expectant mothers with both pre-pregnancy BMI and GWG within standard parameters would exhibit superior prenatal dietary quality.
**Methods**				
Study design	4	Present key elements of study design early in the paper	3	A face-to-face interview was employed to gather demographic information from participants, encompassing age, educational attainment, occupation, parity, and household income.
Setting	5	Describe the setting, locations, and relevant dates, including periods of recruitment, exposure, follow-up, and data collection	3	The participants in this study were expectant mothers who visited the obstetrics outpatient clinic at Jinan Maternal and Child Health Hospital in Shandong Province between January 2021 and December 2022.
Participants	6	(*a*) *Cohort study*—Give the eligibility criteria, and the sources and methods of selection of participants. Describe methods of follow-up<br>*Case-control study*—Give the eligibility criteria, and the sources and methods of case	3	Participants had to meet the following criteria: (1) Chinese citizenship; (2) pregnancy≤12

		ascertainment and control selection. Give the rationale for the choice of cases and controls<br>*Cross-sectional study*—Give the eligibility criteria, and the sources and methods of selection of participants		gestational weeks; (3) have a maternity record established at the institution and have undergone routine obstetric check-ups; (4) face-to-face completion of the survey; (5) plan to give birth at the hospital; and (6) provide signed informed consent. Exclusion criteria included: (1) non-local migrant populations unable to attend routine check-ups; (2) individuals with metabolic disorders or chronic conditions such as tumors or tuberculosis.
		(*b*) *Cohort study*—For matched studies, give matching criteria and number of exposed and unexposed<br>*Case-control study*—For matched studies, give matching criteria and the number of controls per case		
Variables	7	Clearly define all outcomes, exposures, predictors, potential confounders, and effect modifiers. Give diagnostic criteria, if applicable	3-4	A face-to-face interview was employed to gather demographic information from participants, encompassing age, educational attainment, occupation, parity, and household income. The height and weight of the expectant mothers were recorded during their initial antenatal visit, from which the pre-pregnancy BMI was derived. We assessed the dietary quality of expectant

				mothers using the Chinese Healthy Dietary Index for Pregnancy (CHDI-P)
Data sources/ measurement	8*	For each variable of interest, give sources of data and details of methods of assessment (measurement). Describe comparability of assessment methods if there is more than one group	*4*	*Dietary intake data were gathered using a 24-hour dietary recall survey administered over three consecutive days. For the initial survey, both the type and quantity of food consumed were obtained through face-to-face interviews conducted by professionally trained clinicians. To enhance data accuracy, participants were aided in describing their food intake through the utilization of standardized containers (e.g., bowl, spoon, cup) and food imagery supplied by the hospital. To prioritize the safety of the expectant mothers, the subsequent surveys were conducted telephonically. To further refine data accuracy, participants were requested to furnish photographs of all consumed foods for investigator validation.*
Bias	9	Describe any efforts to address potential sources of bias	No	
Study size	10	Explain how the study size was arrived at	no	

Continued on next page

Quantitative variables	11	Explain how quantitative variables were handled in the analyses. If applicable, describe which groupings were chosen and why	4	Descriptive statistics for count data are expressed as numbers and percentages, while continuous variables are represented by means and standard deviations. Between-group comparisons for count data were conducted using the Pearson's $\chi 2$ test. For continuous variables, between-group differences were assessed using analysis of variance (ANOVA) or the Kruskal-Wallis test, contingent upon data normality.
Statistical methods	12	(*a*) Describe all statistical methods, including those used to control for confounding	4	Generalized linear models were employed to adjust for confounding variables when comparing CHDI-P scores across groups. Covariates were selected based on prior research and included age, income, educational level, smoking status, energy intake, number of pregnancies and deliveries (Parker et al., 2019; Shin et al., 2016). Post hoc tests, using the Tukey-adjusted method, elucidated between-group disparities. All confidence intervals were computed at the 95% level, and a p-value less than 0.05 was deemed statistically significant. Statistical analyses were executed using Python 3.6.0.
		(*b*) Describe any methods used to examine subgroups and interactions	no	

		(*c*) Explain how missing data were addressed	no	
		(*d*) *Cohort study*—If applicable, explain how loss to follow-up was addressed<br>*Case-control study*—If applicable, explain how matching of cases and controls was addressed<br>*Cross-sectional study*—If applicable, describe analytical methods taking account of sampling strategy	no	No applicable
		(*e*) Describe any sensitivity analyses	no	
**Results**				
Participants	13*	(a) Report numbers of individuals at each stage of study—eg numbers potentially eligible, examined for eligibility, confirmed eligible, included in the study, completing follow-up, and analysed	5	See table1 and table 2
		(b) Give reasons for non-participation at each stage	no	
		(c) Consider use of a flow diagram	no	
Descriptive data	14*	(a) Give characteristics of study participants (eg demographic, clinical, social) and information on exposures and potential confounders	5	See table1 and table 2
		(b) Indicate number of participants with missing data for each variable of interest	no	
		(c) *Cohort study*—Summarise follow-up time (eg, average and total amount)	no	
Outcome data	15*	*Cohort study*—Report numbers of outcome events or summary measures over time	*5-6*	*See table 3 and 4*
		Case-control study—Report numbers in each exposure category, or summary measures of exposure		
		Cross-sectional study—Report numbers of outcome events or summary measures		
Main results	16	(*a*) Give unadjusted estimates and, if applicable, confounder-adjusted estimates and their precision (eg, 95% confidence interval). Make clear which confounders were adjusted for and why they were included	Table3/4/5/6	See Table3/4/5/6
		(*b*) Report category boundaries when continuous variables were categorized	no	
		(*c*) If relevant, consider translating estimates of relative risk into absolute risk for a meaningful time period	no	

Continued on next page

Other analyses	17	Report other analyses done—eg analyses of subgroups and interactions, and sensitivity analyses	no	
**Discussion**				
Key results	18	Summarise key results with reference to study objectives	6	This study explored the quality of prenatal diets among a convenience sample of pregnant women in Shandong, China. Additionally, we explored the associations between pre-pregnancy BMI, GWG, and maternal dietary quality. While the Alternative Healthy Eating Index for Pregnancy (AHEI-P) has been employed in previous research to quantitatively evaluate the dietary quality of pregnant women (Hsiao et al., 2019; Parker et al., 2019; Quansah et al., 2022). Our study sample was drawn from Shandong, China. Consequently, we utilized the CHDI-P scale, which is better tailored for Chinese pregnant women. Parker et al. determined that using the AHEI-P scale (Parker et al., 2019), the overall prenatal dietary quality of pregnant women was suboptimal, achieving a mean score of just 61.2 out of 130. Similarly, in our study, the average score for pregnant women aligned with this finding. Even after equiproportional conversion, the score indicates that the prenatal

				dietary quality of Chinese pregnant women remains less than ideal. Grouping by GWG did not manifest any significant differences in marginal means among the groups. Conversely, when categorized based on pre-pregnancy BMI, the underweight group had a score that exceeded the obese group by 2.5 points. While a shift of 5% in dietary quality score is necessary to deem it clinically significant(Miller et al., 2015), it is plausible to posit an association between pre-pregnancy BMI and dietary quality. Specifically, pregnant women with a BMI of ⩾25 may be at an elevated risk for malnutrition.
Limitations	19	Discuss limitations of the study, taking into account sources of potential bias or imprecision. Discuss both direction and magnitude of any potential bias	8	This study has some limitations. Firstly, the sample size was limited, primarily because participants were required to deliver at our hospital. This criterion might have introduced some bias. Nonetheless, this was necessary to ensure uniformity across this series of studies. Another notable observation was that pregnant women accompanied by their partners were more inclined to participate and less likely to drop out.

Interpretation	20	Give a cautious overall interpretation of results considering objectives, limitations, multiplicity of analyses, results from similar studies, and other relevant evidence	7-8	Our study examined the association between pre-pregnancy BMI, GWG, and dietary quality. However, a more holistic approach might involve integrating prenatal dietary quality instead of concentrating solely on either BMI or GWG. Both pre-pregnancy BMI and GWG proved valuable in distinguishing and predicting prenatal diet quality. Notably, pre-pregnancy BMI was more sensitive to overall diet quality and restriction of certain foods, whereas GWG was more indicative of dietary adequacy. It's important to highlight that the dietary quality we assessed pertains to the early stage of a woman's pregnancy, while the GWG was determined by the difference between the initial and pre-delivery weights. The dietary habits of pregnant women typically vary throughout the pregnancy. For initial dietary quality assessment, GWG may not be as effective an indicator as BMI, especially in the middle or later stages of pregnancy. This observation warrants further investigation in subsequent research.
Generalisability	21	Discuss the generalisability (external validity) of the study results	8	Our study underscores the

			significance of preconception BMI and GWG as critical factors in predicting prenatal diet quality. While there is a need for further research to validate the relationship between preconception BMI, GWG, and prenatal diet quality, our findings pinpoint pre-pregnancy overweight and obese women as a vulnerable group at heightened risk for suboptimal prenatal diet quality. Future interventions tailored for this group could achieve the most impact by curbing the consumption of unhealthy foods. The data from our study bolster the recommendation for women to attain a normal BMI prior to conception, given its correlation with superior dietary quality.
**Other information**			
Funding	22	Give the source of funding and the role of the funders for the present study and, if applicable, for the original study on which the present article is based	no

*Give information separately for cases and controls in case-control studies and, if applicable, for exposed and unexposed groups in cohort and cross-sectional studies.

Note: An Explanation and Elaboration article discusses each checklist item and gives methodological background and published examples of transparent reporting. The STROBE checklist is best used in conjunction with this article (freely available on the Web sites of PLoS Medicine at http://www.plosmedicine.org/, Annals of Internal Medicine at http://www.annals.org/, and Epidemiology at http://www.epidem.com/). Information on the STROBE Initiative is available at www.strobe-statement.org.
